# Fluoroscopically guided acetabular posterior column screw fixation via an anterior approach

**DOI:** 10.1007/s00064-019-00631-0

**Published:** 2019-10-16

**Authors:** Dietmar Krappinger, Peter Schwendinger, Richard A. Lindtner

**Affiliations:** Department of Trauma Surgery, Medical University of Innsbruck, Anichstraße 35, 6020 Innsbruck, Austria

**Keywords:** Acetabular fracture, Fracture fixation, internal, Posterior hemitransverse fracture, Osseous corridor, Multiplanar reformation, Fluoroscopy, Azetabulumfraktur, Interne Frakturfixierung, Hintere Hemiquerfraktur, Knöcherner Korridor, Multiplanare Rekonstruktion, Fluoroskopie

## Abstract

**Objective:**

Safe posterior column screw fixation via an anterior approach under two-dimensional fluoroscopic control.

**Indications:**

Anterior column with posterior hemitransverse fractures (ACPHF); transverse fractures; two-column fractures and T‑type fractures without relevant residual displacement of the posterior column after reduction of the anterior column and the quadrilateral plate.

**Contraindication:**

Acetabular fractures requiring direct open reduction via a posterior approach; very narrow osseous corridor in preoperative planning; insufficient intraoperative fluoroscopic visualization of the anatomical landmarks.

**Surgical technique:**

Preoperative planning of the starting point and screw trajectory using a standard pelvic CT scan and a multiplanar reconstruction tool. Intraoperative fluoroscopically controlled identification of the starting point using the anterior–posterior (ap) view. Advancing the guidewire under fluoroscopic control using the lateral–oblique view. Lag screw fixation of the posterior column with cannulated screws.

**Postoperative management:**

Partial weight bearing as advised by the surgeon. Postoperative CT scan for the assessment of screw position and quality of reduction of the posterior column. Generally no implant removal.

**Results:**

In a series of 100 pelvic CT scans, the mean posterior angle of the ideal posterior column screw trajectory was 28.0° (range 11.1–46.2°) to the coronal plane and the mean medial angle was 21.6° (range 8.0–35.0°) to the sagittal plane. The maximum screw length was 106.3 mm (range 82.1–135.0 mm). Twelve patients were included in this study: 10 ACPHF and 2 transverse fractures. The residual maximum displacement of the posterior column fracture component in the postoperative CT scan was 1.4 mm (0–4 mm). There was one intraarticular screw penetration and one perforation of the cortical bone in the transition zone between the posterior column and the sciatic tuber without neurological impairment.

## Zusammenfassung

**Operationsziel:**

Sichere Zugschraubenosteosynthese des hinteren Azetabulumpfeilers über einen vorderen Zugang unter zweidimensionaler Bildwandlerkontrolle.

**Indikationen:**

Vordere Pfeilerfrakturen mit hinterer Hemiquerfraktur (ACPHF); Querfrakturen; Zweipfeilerfrakturen und T‑Frakturen ohne relevante Dislokation des hinteren Pfeilers nach Reposition des vorderen Pfeilers und der quadrilateralen Fläche.

**Kontraindikationen:**

Azetabulumfrakturen, die einer direkten offenen Reposition von dorsal bedürfen; sehr enger knöcherner Korridor in der präoperativen Planung; unzureichende intraoperative Darstellung der relevanten Strukturen im Röntgenbildverstärker (BV).

**Operationstechnik:**

Präoperative Planung des Eintrittspunkts und des Schraubenkorridors mithilfe des nativen axialen CT-Datensatzes und eines multiplanaren Rekonstruktionstools. Intraoperative BV-kontrollierte Identifikation des Startpunkts in der a.-p.-Projektion. Vorbringen des Führungsdrahts unter BV-Kontrolle in schräg-seitlicher Projektion. Zugschraubenosteosynthese des hinteren Pfeilers mit kanülierten Schrauben.

**Weiterbehandlung:**

Teilbelastung gemäß den Vorgaben des Chirurgen. Postoperative CT-Kontrolle zur Überprüfung der Schraubenlage und Reposition des hinteren Pfeilers. Keine Implantatentfernung.

**Ergebnisse:**

Der in 100 Patienten bestimmte optimale Schraubenkorridor war durchschnittlich 28,0° (Spanne 11,1–46,2°) nach dorsal (bezogen auf die Koronarebene) und 21,6° (Spanne 8,0–35,0°) nach medial (bezogen auf die Sagittalebene) anguliert. Die maximale Schraubenlänge betrug 106,3 mm (Spanne 82,1–135,0 mm). In diese Studie wurden 12 Patienten eingeschlossen: 10 mit ACPHF und 2 mit Querfraktur. Die durchschnittlich verbliebene Dislokation im Bereich des hinteren Pfeilers betrug 1,4 mm (Spanne 0–4 mm). In einem Fall wurde eine intraartikuläre Schraubenlage und in einem weiteren Fall eine kortikale Perforation im Übergangsbereich zwischen hinterem Pfeiler und Tuber ischiadicum ohne neurologisches Defizit beobachtet.

## Introductory remarks

In acetabular fractures involving one column only, either a single anterior or a single posterior approach is required for internal fixation. However, in acetabular fractures involving both columns, such as transverse fractures, T‑type fractures, anterior column with posterior hemitransverse fractures and two-column fractures, various strategies for surgical stabilization may be considered. Some of these fractures may be treated via a single approach without plate fixation of the other column, while others may require an extended or a combined anterior and posterior approach. The latter increases surgical time, blood loss and morbidity due to the need for a second approach [1–4].

**Fig. 1:**
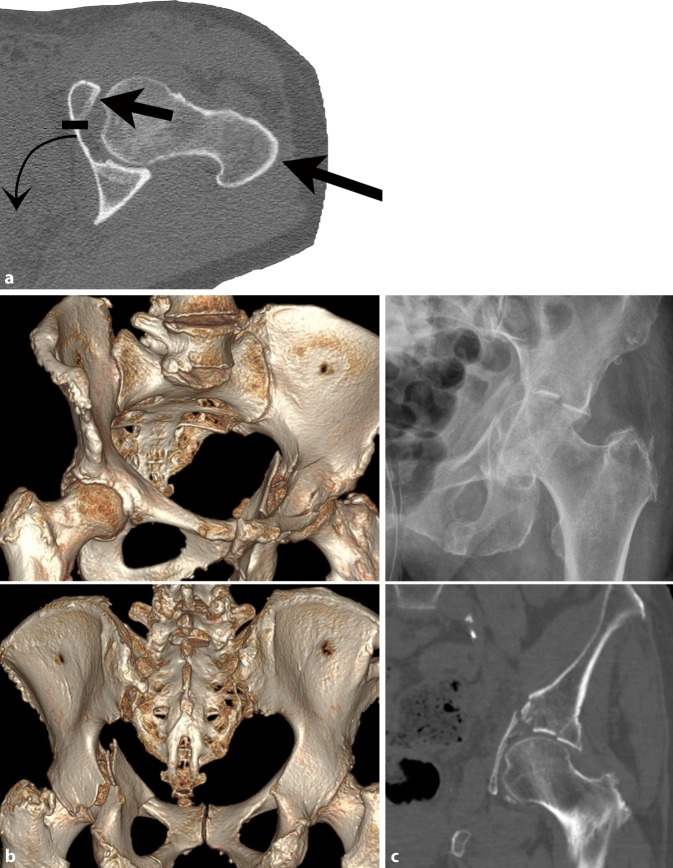
**a** Injury mechanism of anterior column with posterior hemitransverse fractures (ACPHF). ACPHF typically result from force transmission via the greater trochanter and the femoral neck with the hip joint in extension. Due to the anteversion of the femoral neck the anterior column is affected first and frequently shows a multifragmentary fracture pattern. Further protrusion of the femoral head leads to a simple posterior hemitransverse fracture and a fracture component in the transition zone between the anterior column and the quadrilateral plate. The quadrilateral plate therefore remains in osseous continuity with the posterior column. These two fracture components allow for an internal rotation of the posterior column as a result of the medial protrusion of the femoral head. Accordingly, the quadrilateral plate is not separated from the two acetabular columns. It is internally rotated in osseous continuity with the posterior column. **b** Fracture components of ACPHF. The injury mechanism described in **a** results in the typical fracture patterns of ACPHF with the following fracture components and characteristics: multifragmentary or comminuted anterior column fracture; simple posterior hemitransverse fracture; internal rotation of the posterior column and the attached quadrilateral plate; impaction of the articular surface of the superomedial dome (“gull sign”). **c** “Gull sign”: impaction of the articular surface of the superomedial dome results from force transmission via the femoral head. The radiological appearance of this impaction has been described as “gull sign” referring to children’s style of drawing sea gulls. The “gull sign” is associated with a poor outcome after open reduction and internal fixation of acetabular fractures [13–15]

An alternative option for selected acetabular fractures involving both columns is open reduction and plate fixation of the anterior column via an anterior approach combined with a fluoroscopically controlled lag screw fixation of the posterior column via the same single approach [5–7]. One prerequisite of this technique, however, is that the posterior column fracture is either nondisplaced or adequately reduced after reduction of the anterior column and the quadrilateral plate. As a consequence, T‑type fractures or two-column fractures, in general, are less often amenable to this technique, whereas transverse fractures and anterior column with posterior hemitransverse fractures (ACPHF; Fig. 1) appear to be a favorable indication for this fixation strategy. ACPHF and transverse fractures constitute up to 31% of acetabular fractures in patients aged 55 years and older and about 12% of fractures in younger patients, while T‑type and two-column fractures represent more than one third of fractures in both older and younger patients [8, 9].

Although surgical techniques such as CT-controlled and navigated screw fixation may be capable of reducing the rate of screw malpositioning in pelvic and acetabular surgery [10–12], these techniques are not widely available due to their high costs and skills required. The aim of this article therefore is to present a simple preoperative planning technique for the estimation of the starting point and screw trajectory of acetabular posterior column screw fixation via an anterior approach and to delineate its intraoperative application under fluoroscopic control.

## Surgical principles and objective

Safe posterior column screw fixation via an anterior approach under two-dimensional fluoroscopic control in the following two steps:

### First step.

Preoperative planning of the starting point and screw trajectory for posterior column screw placement via an anterior approach using a standard pelvic CT scan and a commonly available multiplanar reconstruction tool.

### Second step.

Intraoperative fluoroscopically controlled identification of the starting point in the pelvic anterior–posterior (ap) view and advancing the guidewire/screw under fluoroscopic control using a lateral–oblique view in order to prevent intraarticular screw penetration and lesions of the sciatic nerve.

### Advantages

- No expensive planning software required
- No intraoperative CT or navigation system required
- Reduced risk of screw malpositioning
- Posterior column screw placement via an anterior approach (Olerud approach or ilioinguinal approach)
- No additional posterior approach required

### Disadvantages

- Relatively high intraoperative radiation exposure
- Fluoroscopic identification of the anatomic landmarks is mandatory
- Time required for preoperative planning
- More challenging technique compared to navigated techniques

### Indications

- Anterior column with posterior hemitransverse fractures
- Transverse fractures
- Two-column fractures and T‑type fractures without relevant residual displacement of the posterior column after open reduction and fixation of the anterior column and the quadrilateral plate

### Contraindications

- Relevant residual displacement of the posterior column after open reduction and fixation of the anterior column and the quadrilateral plate
- Very narrow osseous corridor according to the preoperative planning
- Insufficient fluoroscopic visualization of the anatomical landmarks, i.e., the hip joint and the sciatic tuber
- Patients with severe obesity

### Patient information

- General surgical risks
- Residual risk of screw malpositioning with intraarticular screw penetration or iatrogenic sciatic nerve injury
- Generally no implant removal

### Preoperative work up

**Fig. 2:**
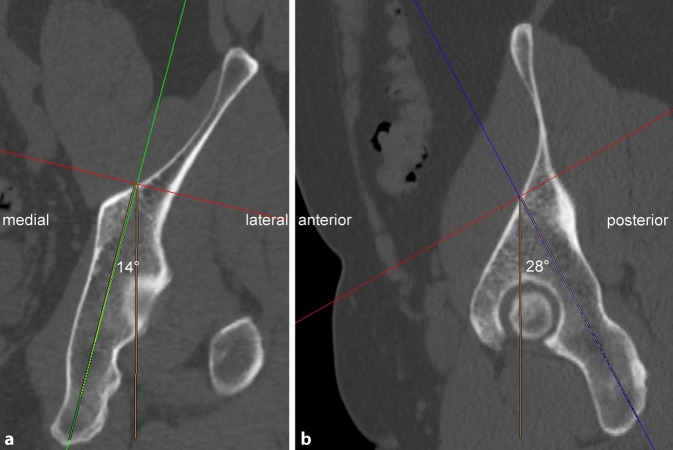
Preoperative assessment of the osseous corridor and ideal posterior column screw trajectory. We highly recommend to assess the ideal posterior column screw trajectory using the uninjured contralateral acetabulum because screw trajectory analysis is easier and more precise in absence of fracture lines and displacement. The uninjured contralateral acetabulum can be reliably used as a template for preoperative planning as the left and right posterior column anatomy (screw insertion angles, screw starting point and screw length) do not significantly differ within the same pelvis (see Results). In case of no or only minor displacement, however, preoperative planning may be also performed on the injured side. The starting point of the posterior column screw is located in the transition zone between the supraacetabular region and the iliac wing on the inner cortex of the iliac bone. The screw trajectory is oriented from cranial–anterior–lateral to caudal–posterior–medial (**a**, **b**). The ideal starting point and screw trajectory are assessed by using native axial CT images and a two-dimensional multiplanar software reconstruction tool. Therefore, the axes of coordinates are translated and the axes itself rotated to assess the ideal entry point and screw trajectory. In the present case, the medial angle of the ideal posterior column screw trajectory is 14° to the sagittal plane (**a**) with a maximum screw length of 130 mm from the starting point to the end point (cortex of the sciatic tuber). Furthermore, the posterior angle of this screw trajectory is 28° to the coronal plane (**b**) and obviously shows the same maximum screw length in this second reformation plane

**Fig. 3:**
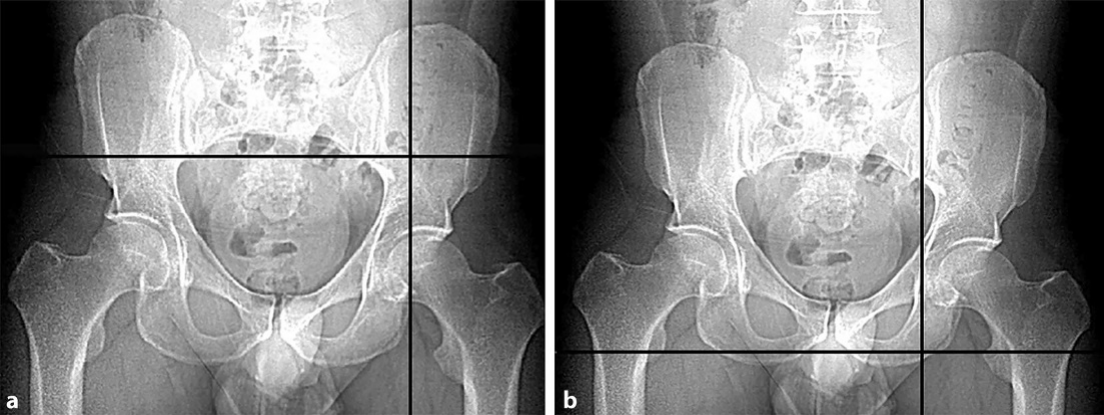
Estimation of the starting point and endpoint of the ideal screw trajectory. This is one of the major challenges in fluoroscopically controlled posterior lag screw fixation. The preoperative planning is performed using multiplanar CT reformations in all three dimensions. The intraoperative application, however, is performed using two-dimensional (2D) fluoroscopic control. The CT scout image is used to facilitate this transition. It is a low-dose pelvic X‑ray routinely obtained during standard CT imaging, which is usually performed in a standard supine position and thus corresponds to intraoperative positioning of the patient. Pads or pillows supporting the lumbosacral region modify the pelvic tilt and should therefore not be used intraoperatively. Software tools allow for a real-time localization of any arbitrary CT point in the scout view (“LiveSync” feature). Thus, the surgeon is able to transfer the ideal screw starting (**a**) and endpoint (**b**) identified in multiplanar CT reformations to the 2D anterior–posterior (ap) view scout image. This greatly facilitates identification of the ideal localization of the screw starting and end point (and therefore of the screw trajectory) in the intraoperative pelvic ap view

The preoperative planning is the major step for safe fluoroscopically controlled posterior column screw placement and is therefore described in detail (Fig. 2 and 3). Pelvic CT scans with a slice depth of 0.6 mm are recommended for preoperative planning. CT scans with slice depths >0.6 mm are also applicable, but may result in inferior image quality during the reformation process. Any imaging software, which supports two-dimensional multiplanar reformation (MPR), is suitable for preoperative assessment.

### Instruments and implants

- Guide wire (2.8 mm diameter)
- Partially threaded cannulated large fragment screws (16 mm or 32 mm thread) for the application as lag screws

### Anesthesia and positioning

- General anesthesia
- Supine positioning

## Surgical technique

Figs. 4, 5, 6

**Fig. 4:**
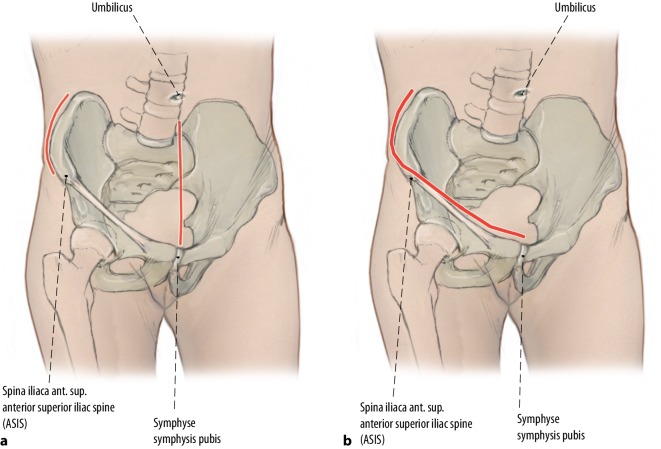
Modified Stoppa combined with Olerud approach vs. ilioinguinal approach. Open reduction of the anterior column is performed first via either a modified Stoppa approach (**a**) or an ilioinguinal approach (**b**). In transverse fractures, the single fracture line is addressed from anterior. In anterior column with posterior hemitransverse fractures (ACPHF), the posterior hemitransverse fracture is reduced by reduction of the quadrilateral plate, which is attached to the posterior column as outlined above. After reduction and anterior column fixation, the posterior column screw fixation is performed via the lateral window of the ilioinguinal approach. If a modified Stoppa approach is used, this approach is frequently combined with an Olerud approach for anterior column fixation (**a**). In this case, the posterior column lag screw can be easily placed via the Olerud approach. If only a modified Stoppa approach is used for the fixation of the anterior column, a second small incision at the iliac crest (“Mini Olerud approach”) is applied to approach the entry point

**Fig. 5:**
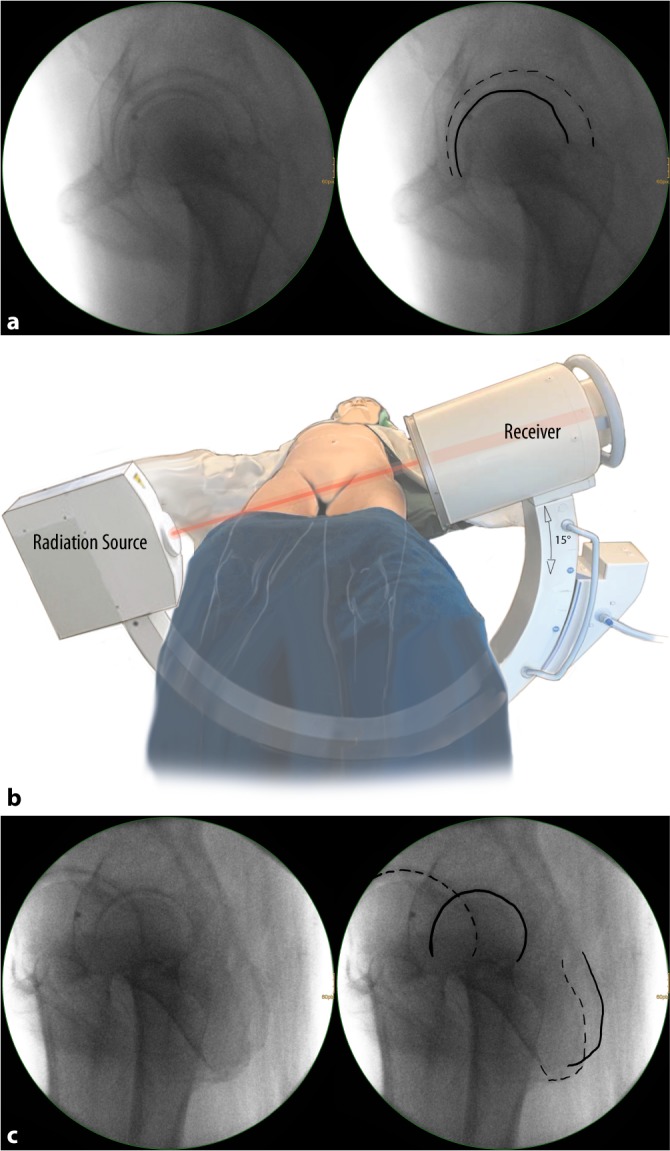
**a** Lateral view of the hip. Two views are mandatory for fluoroscopically controlled posterior column screw fixation. The anterior–posterior (ap) view is a standard view, which allows for the assessment of the entry point according to preoperative planning. The second view is a lateral–oblique view (**b** and **c**). In general, the lateral view of the hip (**a**) is not very commonly used, as it is inevitable that both hips are projected into each other. The magnification effect, however, allows for a simple differentiation between the two hips. The “larger” hip (*dotted line*) is located near the radiation source, while the “smaller” hip (*solid line*) is located near the receiver of the C‑arm. **b** C-arm and patients’ adjustments for the lateral–oblique view of the hip. The C‑arm is tilted approximately 15° for the lateral–oblique view of the hip. The hip joints are internally rotated to prevent fluoroscopic projection of the femoral neck and the greater trochanter onto the posterior column. **c** Lateral–oblique view of the hip. A lateral–oblique view with the radiation source on the right side and the receiver tilted 15° upwards on the left side is shown. The left hip and posterior column (*solid lines*) are located near the receiver and are therefore “smaller” than the right-sided structures (*dotted lines*). The left hip is also projected more posteriorly due to the tilt of the C‑arm

**Fig. 6:**
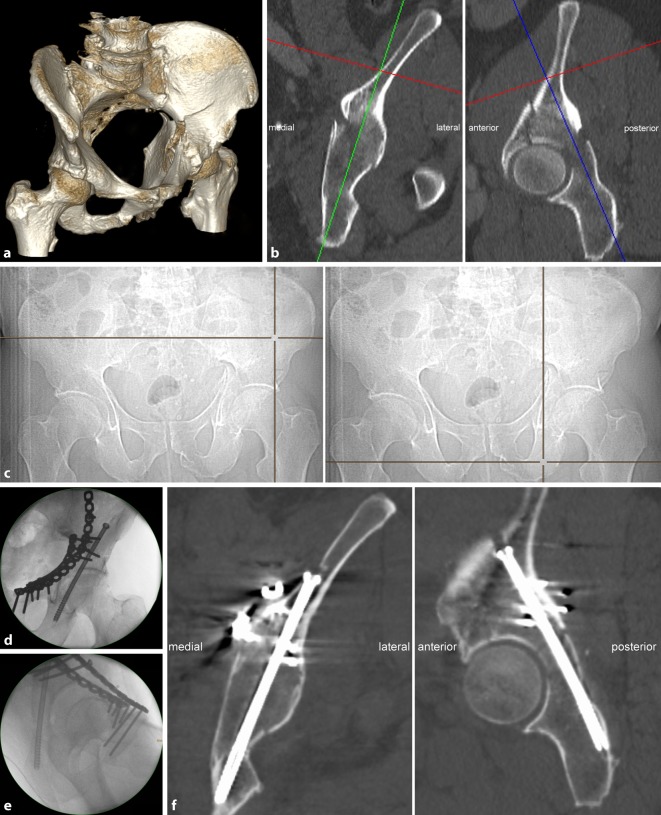
**a** Posterior column screw—fracture type. A 67-year-old man who sustained a bicycle accident with a direct impact on the left hip. The CT scan shows an anterior column with posterior hemitransverse fractures (ACPHF) with a multifragmentary fracture of the anterior column. The quadrilateral plate is internally rotated and in osseous continuity with the posterior column. The posterior hemitransverse fracture is simple as shown in **b**. **b** Preoperative planning—posterior column screw trajectory. The medial angle of the posterior column screw trajectory is 16° to the sagittal plane (*left*) and the posterior angle is 24° to the coronal plane (*right*). The maximum screw length is 123 mm. **c** Preoperative planning—estimation of the starting point and the endpoint. The starting point and the end point are determined using the LiveSync feature as described in Fig. 3. **d** Intraoperative application—fluoroscopic control in anterior–posterior (ap) view. The anterior column fracture was reduced via a modified Stoppa approach and stabilized with two 3.5 pelvic reconstruction plates. The posterior hemitransverse fracture was indirectly reduced by reducing the quadrilateral plate. The starting point for the posterior column screw was approached via a small incision at the iliac crest and submuscular preparation under fluoroscopic control in the ap view. The guide wire was advanced according to preoperative planning (**b**). It is advisable to switch to the lateral–oblique view when approaching the hip joint. After predrilling, a cannulated 6.5 mm screw with a 32 mm thread and a length of 115 mm was inserted. The screw length must not be longer than the maximum length determined in preoperative planning, but may be shorter provided that the screw thread completely passes the fracture line. The fluoroscopic control shows a screw trajectory as preoperatively planned (**c**). **e** Intraoperative application—fluoroscopic control in lateral–oblique view. The left hip joint is located next to the radiation source which results in a “larger” left hip joint. Additionally, the radiation source is tilted 15° upwards resulting in a more posterior projection of the left hip. The lateral–oblique view shows that the screw does not penetrate the hip joint and does not perforate the cortical bone of the posterior column and the sciatic tuber. **f** Postoperative CT control. The correct screw trajectory is confirmed in the postoperative CT scan. The posterior hemitransverse fracture is anatomically reduced

### Postoperative management

- Partial weight bearing as advised by the surgeon
- Postoperative CT imaging for the assessment of screw position and quality of reduction of the posterior column is recommended
- Generally no implant removal

### Errors, hazards and complications

**Fig. 7:**
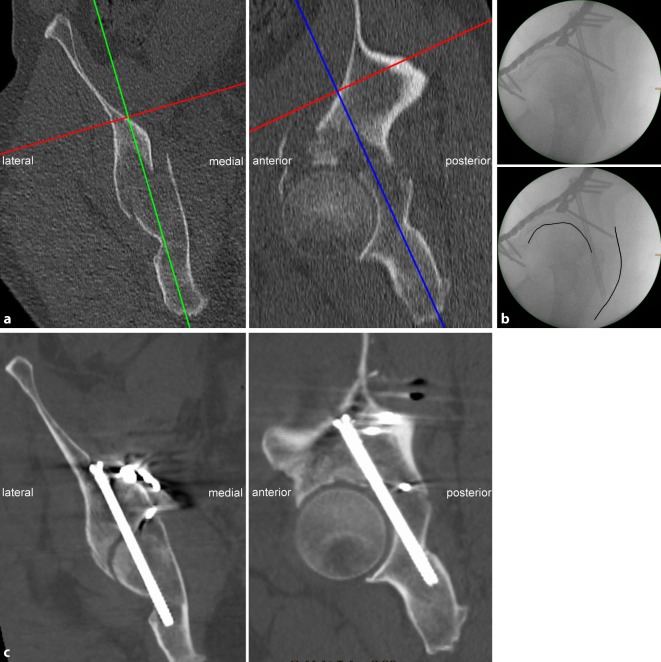
Screw misplacement. **a** Preoperative planning for a 56-year-old man who sustained a typical anterior column with posterior hemitransverse fracture (ACPHF) on the right side. The preoperative planning reveals a relatively narrow osseous corridor for a posterior column screw. **b** Fluoroscopic control in lateral–oblique view. The image quality is moderate in general. Additionally, the lateral–oblique view was not sufficiently oblique resulting in a poor differentiation between the left and the right hip and sciatic tuber (*upper image*). The screw does not perforate the cortical bone at the sciatic tuber, but is tangent to the projection of the hip joint line (*lower image*). **c** Postoperative CT scans revealed adequate reduction of the posterior hemitransverse fracture via the quadrilateral plate. The posterior column screw, however, penetrates the cortex of the acetabular fossa. The hip joint motion was not restricted and the patient refused revision surgery

- Inadequate reduction of the posterior hemitransverse fracture via an anterior approach: open reduction via a posterior approach
- Poor fluoroscopic visualization of the anatomical landmarks
- Narrow osseous corridor
- Screw misplacement: revision surgery in the case of neurological impairment or intraarticular screw penetration (Fig. 7)

## Results

Osseous corridors and resulting ideal screw trajectories for posterior column screws were assessed in a series of 100 pelvic CT scans. These CT scans were performed during clinical routine with the indications for imaging not related to this study. Accordingly, the patients were not exposed to additional radiation. There were 50 female and 50 male patients with a mean age of 57.0 years (range 18–90 years). The mean posterior angle of the ideal posterior column screw trajectory was 28.0° (range 11.1–46.2°) to the coronal plane and the mean medial angle was 21.6° (range 8.0–35.0°) to the sagittal plane. The maximum screw length was 106.3 mm (range 82.1–135.0 mm). There were no significant differences between the right and the left side (t-test for paired samples, *p* > 0.05). There were also no significant differences between male and female patients regarding the posterior and medial angles of the ideal posterior column screw trajectories (t-test for independent samples, *p* > 0.05). The average maximum screw length, however, was significantly greater in male patients (111.9 ± 9.3 mm vs. 99.7 ± 8.5 mm, *p* < 0.01).

Twelve patients were included in this study. The small number of patients is attributable to the fact that only a subset of acetabular fractures involving both columns is amenable to posterior column fixation via an anterior approach. Additionally, only patients with postoperative CT scans available for review were included. There were 10 ACPHF and 2 transverse fractures. The residual maximum displacement of the posterior column fracture components was 1.4 mm (range 0–4 mm). Beside the case shown in Fig. 7, there was one case with a perforation of the cortical bone in the transition zone between posterior column and sciatic tuber (maximum screw protrusion of 5 mm) without neurological impairment.

## References

[1] Gänsslen A, Krettek C (2009). Internal fixation of acetabular both-column fractures via the ilioinguinal approach. Oper Orthop Traumatol.

[2] Ochs BG, Marintschev I, Hoyer H (2010). Changes in the treatment of acetabular fractures over 15 years: analysis of 1266 cases treated by the German Pelvic Multicentre Study Group (DAO/DGU). Injury.

[3] Jeffcoat DM, Carroll EA, Huber FG (2012). Operative treatment of acetabular fractures in an older population through a limited ilioinguinal approach. J Orthop Trauma.

[4] Tosounidis TH, Giannoudis PV (2015). What is new in acetabular fracture fixation?. Injury.

[5] Shahulhameed A, Roberts CS, Pomeroy CL (2010). Mapping the columns of the acetabulum—implications for percutaneous fixation. Injury.

[6] Chen W, Zhang Z, Lu Y (2014). Fluoroscopic views for safe insertion of lag screws into the posterior column of the acetabulum. BMC Musculoskelet Disord.

[7] Osterhoff G, Amiri S, Unno F (2015). The “Down the PC” view—a new tool to assess screw positioning in the posterior column of the acetabulum. Injury.

[8] Daurka JS, Pastides PS, Lewis A (2014). Acetabular fractures in patients aged >55 years: a systematic review of the literature. Bone Joint J.

[9] Ferguson TA, Patel R, Bhandari M (2010). Fractures of the acetabulum in patients aged 60 years and older: an epidemiological and radiological study. J Bone Joint Surg Br.

[10] Peng KT, Li YY, Hsu WH (2013). Intraoperative computed tomography with integrated navigation in percutaneous iliosacral screwing. Injury.

[11] Fischer S, Vogl TJ, Marzi I (2015). Percutaneous cannulated screw fixation of sacral fractures and sacroiliac joint disruptions with CT-controlled guidewires performed by interventionalists: single center experience in treating posterior pelvic instability. Eur J Radiol.

[12] Richter PH, Gebhard F, Dehner C (2016). Accuracy of computer-assisted iliosacral screw placement using a hybrid operating room. Injury.

[13] Anglen JO, Burd TA, Hendricks KJ (2003). The “Gull Sign”: a harbinger of failure for internal fixation of geriatric acetabular fractures. J Orthop Trauma.

[14] Laflamme GY, Hebert-Davies J, Rouleau D (2011). Internal fixation of osteopenic acetabular fractures involving the quadrilateral plate. Injury.

[15] Krappinger D, Lindtner RA, Resch H, Blauth M, Kates SL, Nicholas JA (2018). Acetabulum. Osteoporotic fracture care: medical and surgical management.

